# Progress in Surgery of the Pancreas and Spleen

**Published:** 1903-02-21

**Authors:** 


### PROGRESS IN SURGERY OF THE PANCREAS AND SPLEEN.

**Chronic Pancreatitis**, according to Moynihan[1], was scarcely recognised until Riedel first called attention to the association of thickening of the head of the pancreas with cholelithiasis. Chronic pancreatitis as seen by the surgeon is always secondary. It is dependent upon infection extending from the intestinal canal or from the bile passages upon the long-standing irritation of gall or pancreatic stones, or upon the invasion of malignant disease, which may be primary in the gland itself or may extend into the gland from the duodenum or stomach. Certain toxic substances brought by the blood to the gland may set up chronic inflammation; of such are the poison of syphilis, the organism of tubercle and alcohol. Pre-eminent among all these causes we must place cholelithiasis. Associated with, and dependent upon, gall-stone disease may be found every step in the extension of inflammation to the gland. In the slightest cases a little thickening of the head of the gland may be found; that portion nearer to the duodenum is denser and stiffer than the body or tail. In the severer cases the whole head and a part of the body also may be enlarged and extremely hard and solid, feeling like a plaster cast of an enlarged gland. But in all cases the duodenal end is more profoundly affected by the changes which have taken place than is the body. The tail is implicated only in the long-standing and severer instances. Opie distinguishes two forms of interstitial inflammation of the gland—interlobular, in which the inflammatory process is localised chiefly at the periphery of the lobule; and interacinar, in which the process is diffuse, invading the lobules and separating individual acini and involving the islands of Langerhans. The former is the more common. The distinction between the two forms is important, for in the interlobular variety the islands of Langerhans are rarely affected, and then only in the latest stages, and therefore diabetes is exceptional. Syphilitic disease of the pancreas may assume two forms, a chronic diffuse interstitial inflammation, or a localised gummatous inflammation. It is more common in children than in adults, and is frequently a congenital lesion. The disease begins as a periarteritis. The treatment of chronic pancreatitis must be mainly concerned with the removal of the cause of the gland implication. But in all cases drainage of the gall-bladder is necessary. Owen,[2] in reporting two cases, says that as it is often quite impracticable to distinguish chronic inflammation of the pancreas from cancer, and as abdominal section is in some strange way the means of cure in the case of inflammatory disease, it is surely advisable that an exploratory operation should be undertaken in all cases of obscure disease in the cœliac region. Operative interference has shown that drainage of the gall-bladder does not always cure chronic pancreatitis, and also that the disease may get well without it. Owen therefore asks the question whether drainage of the gall-bladder is a necessary part of the operative treatment of chronic pancreatitis.

**Rupture of the Spleen.**—Sheild[3] adds another case to the series of successful splenectomies for injury. The case is principally of interest as showing what desperate lesions may be recovered from by prompt abdominal surgery. In some cases of rupture of this organ the tear is slight and superficial, and such cases may be treated by the gauze tampon or by suture of the splenic substance. In the present case the laceration of the organ was so extreme that it was wonderful that the patient did not die from hæmorrhage. The abdomen was full of blood and the clamping of the pedicle was done entirely by feeling, it being impossible to clear the area by sponging. In cases where the symptoms of rupture of the spleen are marked probably abdominal section gives the best chance. Violent tearing pain and local swelling indicate persistent and furious bleeding and the need for speedy operation. Large and dangerous anomalous vessels, such as are met with in cases of splenic leukæmia and other conditions where the spleen is much enlarged, are not so frequent or perilous in removal of a spleen of normal size which may be extensively ruptured. In minor ruptures of the spleen the extravasation of blood is more gradual, and the surgeon may wait until the primary shock has passed away. It is in these cases that the gauze tamponade is so useful, or actual suture of splenic substance with catgut. As a general truth it may be stated that exploration where signs of grave internal lesions are evident and persistent is becoming more and more the practice of the day in English hospitals.

[1] Lancet, Sept. 27, 1902, p. 856. [2] Brit. Med. Jour., Oct. 25, 1902, p. 1311. [3] Lancet, Oct. 25, 1902, p. 1125.

